# The Story of the Insane from Year to Year

**Published:** 1900-12-29

**Authors:** 


### THE STORY OF THE INSANE FROM YEAR TO YEAR.

(Thirteenth Series.)

### THE CITY OF LONDON ASYLUM AT STONE, NEAR DARTFORD.

This asylum, or hospital for mental diseases, contained 478 patients on January 1st, 1899; and at the end of December the number had fallen to 460. There were 142 first admissions, 10 readmissions, 66 recoveries, 67 discharged relieved, and 36 deaths. The recoveries stand at the very high percentage of 57·89 of the admissions, and the deaths at the low percentage of 7·42 on the average number resident. Of the deaths 16 were caused by cerebral and spinal diseases, 1 by phthisis, 1 by pneumonia, and one by enteritis. Of the year's admissions 23 were driven insane by moral causes (domestic troubles, mental anxiety, religion, &c.), 36 by intemperance in drink, 39 by previous attacks, and 43 by hereditary influence. As already stated there was only one death from pulmonary consumption; and Dr. White informs us that since the opening of the asylum in 1866 the average death rate from this disease has only been 9·32 per cent. of the total number of deaths, whereas in English county and borough asylums it has been 15·47 per cent. This is a very remarkable fact and we should much like to hear whether Dr. White has any explanation of it. There was one death from colitis, and there were several non-fatal cases of enteric fever. As the sanitary arrangements are now in an excellent state, it is hoped that these diseases will entirely disappear. Nursing lectures have been given in this asylum for a great many years, and those of the staff who hold nursing certificates are allowed extra pay. A great many private patients are received in this asylum, and the charge is 21s. a week each. This proceeding is not only a source of profit to the asylum but it is a real boon to many a patient who cannot afford to pay the charges made in private asylums, and who do not like to incur the stigma of receiving "charity" in a lunatic hospital although they may have to pay more than the sum charged at the Stone Asylum. The committee say that they "have pleasure in recording that the medical superintendent continues to display zeal and ability in the discharge of his duties." The weekly charge for the city patients is 11s. 1d. per head.

### THE LIMERICK DISTRICT ASYLUM.

At this asylum 629 patients were in residence on the first day of 1899, and at the end of the year the number was reduced to 622. There were 104 first admissions, 30 readmissions, 55 recoveries, 16 discharged relieved, and 69 deaths. The recoveries stand at 41 per cent. of the admissions, and the deaths at 11 per cent. of the average number resident. Of the deaths 12 were caused by cerebral and spinal diseases, 27 by consumption, and 6 by pneumonia. The insanity in the admissions of the year was induced by moral causes in 18 instances, by intemperance in drink in 19, and by hereditary influence in 22. Dr. O'Neill says, when speaking of the death-rate, that until the spread of phthisis "is prevented by isolation in the early stage of the disease, it will continue to swell the death-rate of our asylums, no matter how favourable their hygienic condition may be, and, unfortunately, its low lying site aud overcrowded condition of this asylum for many years past have tended in a marked degree to the development of the disease." To obtain detached hospitals for phthisis at our county asylums will, we fear, be a work of considerable time, but perhaps something could be done, even at an asylum so unfavourably circumstanced as Limerick, to reduce the death-rate. For instance, fresh air out of doors by day, pure air in dormitories at night, isolation of the worst cases in a separate ward, and an improved, or altered, diet in all. New additions were opened during the year, and it is anticipated that alterations will be made in the administrative department ere long. Dr. O'Mara, the assistant medical officer, left on being appointed superintendent of the Ennis Asylum, and Dr. Coffey fills his post at Limerick. The matron retired on her pension, and was succeeded by Miss Cregan. The average cost per head was £21 5s. 1d. per annum.

### THE KENT COUNTY ASYLUM AT CANTERBURY.

THIS asylum contained 1,125 patients on January 1st, 1899, and the number had risen to 1,160 at the end of the year. There were 279 admissions, 115 recoveries, 12 discharged relieved, and 98 deaths. The recoveries reached 43 per cent. of the admissions, and the deaths stood at 8 per cent. of the average number resident—the former being distinctly above the usual asylum average, and the latter below it. Of the deaths 26 were caused by cerebral and spinal diseases, 18 by consumption, 6 by pneumonia, 5 by lung congestion, 7 by bronchitis, and 4 by colitis. Of the admissions moral causes account for the insanity in 53 instances, intemperance in drink in 53, influenza in 9, and hereditary influence in 85. Additions were lately put up at this asylum, and already they are filled or almost so; but with the discharge of the London cases from the Barming Heath Asylum a considerable number of beds will be free. There were 30 cases of colitis and, as already stated, four deaths. The outbreak is attributed to a faulty drain. Ten of the staff obtained the Nursing Certificate of the Medico-Psychological Association, showing that Dr. FitzGerald has continued the course of lectures and instruction. The weekly rate is 9s. 10⅝d.

### THE BALDOVAN ASYLUM FOR IDIOT CHILDREN, DUNDEE.

THIS asylum contained 100 patients on January 1st, 1899, and 102 on December 31st. There were 26 admissions, 4 discharged relieved, 4 not improved, and 15 died. The average residence of those who were discharged was three years. Of the deaths 7 were due to tuberculosus disease, 3 to epilepsy, and 2 to hydrocephalus. The old building is looked upon as unsatisfactory in several ways, and a new asylum is being built on a much finer site. The cost is estimated at something over £9,000.

### THE LEICESTER BOROUGH ASYLUM.

ON January 1st, 1899, this asylum contained 552 patients, and on December 31st the number was 571. There were 91 first admissions, 27 readmissions, 57 recoveries, 8 discharged relieved, and 30 deaths. The recoveries stand at 50 per cent. of the admissions, and the deaths at 5·3 per cent. on the average number resident. The recovery rate is very high, and the death rate is very low. Of the deaths, 11 were caused by cerebral and spinal disease, 2 by phthisis, 1 by congestion of the lungs, and 1 by pneumonia. Of the year's admissions, 11 owed their insanity to moral causes (domestic trouble, adverse circumstances, &c.), 10 to intemperance in drink, 31 to previous attacks, 33 to hereditary influence, and in 40 no cause was known. Such a proportion of "unknown" militates greatly against the usefulness of these statistics, and we think the number might have been reduced by inquiries put by the relieving officers previous to admission, or by the asylum medical officers afterwards, although it hardly comes within the medical officer's duty to make these inquiries. There was no epidemic disease, and, as already stated, the death-rate was remarkably low. It is curious to notice how very much the death-rate from consumption varies in our county and borough asylums. Here the daily average number of patients resident was 561, and there were only two deaths from that disease; whereas in another asylum we lately noticed there were twenty-seven deaths from phthisis in a total daily average number resident of 629 patients. Additions to this asylum were begun in 1897, and yet we are told that the centre block has not advanced beyond the foundations, and that there still remains a large amount of woodwork and other fittings to be done in the main structure. Such delay is extraordinary in the face of Dr. Finch's words that "it becomes more and more difficult to find accommodation for the increasing number of patients." The committee record "their appreciation of the highly satisfactory manner in which Dr. Finch and the officers of the institution generally have discharged their respective duties. The weekly cost per head was 10s. 4⅞d.

### THE MONOGHAN DISTRICT ASYLUM.

THE limits of accommodation are stated to be for 723 patients, but there were 760 in residence on January 1st, 1899, and by the end of the year the number had risen to 778. There were 118 first admissions, 27 re-admissions 72 recoveries, 12 discharged relieved, and 41 deaths. The recoveries stand at the high percentage of 49·7 calculated on the admissions, and the deaths calculated on the average number resident are 5·4 per cent.—a very low death rate. Of the deaths 14 were due to cerebral and spinal diseases, 8 to consumption, and 1 to pneumonia. Of the year's admissions 17 are supposed to have owed their insanity to various moral causes, 12 to intemperance in drink, and 35 to hereditary influence, while in 48 there was no cause known. As usual in asylums in Ireland the visiting committee present no report, or at least do not publish one, and it happens that Dr. Taylor's report is very short. It would seem, however, that a new chapel was opened during the year, and that a considerable part of the asylum has had a heating apparatus fitted up which works satisfactorily. The Inspector's report tells us that the overcrowding, which has been noticed year after year, continues, and that even the temporary buildings were not occupied on the day of his visit. The medical officers give instructions in nursing to the staff, and 5 attendants and 4 nurses hold the certificate of the Medico-Psychological Association. The cost per head per annum was £22 2s.

